# Perspectives on Involving Patients in the Teaching and Assessment of Entrustable Professional Activities in Competence by Design

**DOI:** 10.1111/tct.70129

**Published:** 2025-06-16

**Authors:** Holly Adam, Kaylee Eady, Katherine A. Moreau

**Affiliations:** ^1^ Faculty of Education University of Ottawa Ottawa Ontario Canada

**Keywords:** competence by design, competency‐based medical education, entrustable professional activities, EPA assessment, EPA teaching, patient involvement

## Abstract

**Background:**

Involving patients in teaching and assessing entrustable professional activities (EPAs) within competency‐based medical education (CBME) enhances the authenticity of postgraduate medical education and helps develop learners' skills. However, CBME, including Competence by Design in Canada, lacks clear guidance on involving patients. This study examines faculty members' perspectives on involving patients in teaching and assessing EPAs.

**Methods:**

We conducted semistructured interviews with 25 faculty members from 14 Canadian medical schools across eight residency specialties. We asked participants how they envisioned patients being involved in teaching and assessing EPAs during EPA creation, how they thought patients could contribute to teaching and assessing EPAs, and what they perceived as barriers. We analysed the data thematically.

**Findings:**

Faculty members view patients as subjects for teaching EPAs rather than as teachers of EPAs. However, they noted that patients can teach aspects of EPAs through storytelling, if provided the opportunity. They recognized the value of patients as assessors, particularly in formative assessments of learners' nontechnical skills embedded within EPAs. Nevertheless, they expressed concerns about patient involvement in summative assessments and a lack of EPAs focusing on skills that patients can assess. They noted several barriers to involving patients in teaching and assessing EPAs.

**Conclusion:**

Our study underscores the pressing need for change and the crucial role of more explicit guidance on involving patients in teaching and assessing EPAs. This is not just a matter of academic interest, but a step towards enhancing the quality of medical education.

## 1 Introduction

Involving patients in postgraduate medical education adds realism to learning, helps learners retain information, and encourages learners to reflect on their interactions with patients [1, 2, 3]. It also strengthens learners' nontechnical skills (i.e., the cognitive, social, and personal skills that work together to contribute to quality health care and effective physician‐patient interactions) [4]. In competency‐based medical education (CBME), entrustable professional activities (EPAs) are the foundation for teaching and assessing learners. Patients can teach and assess EPAs. They can help teach residents the skills and professional behaviours required to perform EPAs independently and safely [3, 4, 5, 6, 7]. Patients also have an experiential understanding of patient‐physician interactions and directly observe learners. Thus, they can contribute to learners' EPA assessments by providing input to competence committees (i.e., groups of medical educators responsible for making high‐stakes decisions about a learner's progress on EPAs) [6, 7, 8].

**What are EPAs?**

EPAs are activities that physicians do every day [9]. EPAs progress from basic to complex skills as learners transition through their training. Within each EPA, there are milestones. Milestones are specific skills that trainees need to demonstrate to accomplish the EPA. An example of an EPA for Obstetrics and Gynaecology is: *Providing routine prenatal care to a low‐risk, healthy population* [10]. One milestone for this EPA is: *Address the patient's ideas, fears, and concerns about pregnancy and her prenatal care*.

The Royal College of Physicians and Surgeons of Canada (RCPSC) recognizes that patients can be involved in teaching and assessing EPAs through, for example, multisource feedback [11]. They also endorse a Charter that outlines principles for implementing CBME effectively. This Charter acknowledges that patients can define the competencies taught in EPAs and provides guidelines for their involvement [12]. However, the extent to which residency programs involve patients in teaching and assessing EPAs is unclear. Research on CBME primarily focuses on its implementation rather than patient involvement. Therefore, there is little guidance on involving patients in teaching and assessing EPAs [13, 14]. Faculty members have expressed uncertainty about how to initiate patient involvement, who should take responsibility for it, and how to prepare patients for teaching and assessment roles [15].

This study examines faculty members' perspectives on the inclusion of patients in teaching and assessing EPAs from the Canadian hybrid model of CBME known as Competence by Design (CBD). The following research questions guided this study:

1. During the creation of EPAs, how were patients envisioned to be involved (or not involved) in teaching and assessing EPAs?
2. How can patients contribute to teaching and assessing EPAs?
3. What are the perceived barriers to involving patients in teaching and assessing EPAs?

## 2 Materials and Methods

Ethics approval was obtained from the University of Ottawa Research Ethics Board (REB # S‐02‐22‐7832). We purposefully recruited faculty members involved with EPAs (i.e., in creating, teaching, and assessing EPAs) across a random sample of eight residency specialties (i.e., General Internal Medicine, Obstetrics and Gynaecology, Psychiatry, Anesthesiology, Radiation Oncology, General Surgery, Emergency Medicine and Physical Medicine and Rehabilitation) in 14 English‐speaking medical schools across Canada [16]. We recruited participants in three rounds. In Round 1, we emailed 146 program directors and recruited 5 participants. In Round 2, we contacted 206 involved with EPAs and recruited 10 more. In Round 3, we sent 177 follow‐up emails to those who did not respond in Rounds 1 or 2, resulting in 10 additional participants. Overall, we sent 529 recruitment emails.

We conducted one‐on‐one semi‐structured virtual (via Zoom) interviews with each participant, lasting 30–60 min. We asked about their experiences and perspectives on involving patients in teaching and assessing EPAs. Interviews were audio‐recorded and transcribed verbatim. We inductively and thematically analysed the data [17]. Specifically, we (HA and KM) independently read each transcript several times and extracted quotes that answered the above‐mentioned research questions. Then, we clustered quotes with similar messages and ideas, read each cluster several times, and determined overarching themes. To improve the trustworthiness of our analyses, we each wrote analytic memos, which acted as audit trails of our decision‐making processes [18]. Finally, we discussed our decision‐making, compared and finalized the themes, and agreed on exemplar quotes to include.

## 3 Findings

### 3.1 Participant Characteristics

Twenty‐five individuals participated in this study; all were involved in teaching and assessing EPAs, and 14 were also involved in creating them. Participants from each specialty included three from General Surgery, two from Anesthesiology, two from Psychiatry, seven from Emergency Medicine, four from General Internal Medicine, four from Obstetrics and Gynaecology, two from Physical Medicine and Rehabilitation, and one from Radiation Oncology.

## 4 During the Creation of EPAs, How Were Patients Envisioned to be Involved (or Not Involved) in Teaching and Assessing EPAs?

Participants described that patients were envisioned to be involved as (1) subjects for teaching EPAs rather than teachers of EPAs and (2) formative assessors of EPAs.

### 4.1 Subjects for Teaching EPAs Rather Than Teachers of EPAs

Participants involved in EPA creation explained that ‘there was almost no discussion’ (P7, Gen. Surg.) about how patients could actively teach EPAs. For example, participants remarked, ‘Were patients discussed in teaching? Not to my recollection’ (P9, Psychiatry). Instead, during the creation of EPAs, the participants described how the EPA creators envisioned patients as subjects for teaching EPAs rather than teachers of EPAs. They justified patients as subjects rather than teachers because patients' needs, expectations, and insights are at the heart of each EPA:

We did not really talk too much about patients [in teaching EPAs]. In the establishment of objectives, we used CanMEDS, and we leaned on that quite a bit because there's a big public input into that. Each CanMEDS iteration has public involvement, so we used that as a stepping‐stone to establish our EPAs. (P5, OBGYN)

They assumed that because EPAs are inherently ‘specific to patient scenarios, … are very patient focused’ (P3, ANESTH), and ‘[are] focused on patient care’ (P12, PM&R), there is no need for EPAs to be taught by patients. They further argued that EPAs are ‘too technical’ (P1, Gen. Surg.) for patients to teach:

We talked about mostly technical things … [and] that [would] require [patient] commitment, understanding of the [teaching] process. (P1, Gen. Surg.)

### 4.2 Formative Assessors of EPAs

Participants involved in creating EPAs discussed how they envisioned patients assessing EPAs through 360° evaluations and multisource feedback. They acknowledged that patient assessments are ‘an adequate and reasonable source’ (P3, ANESTH) that should be collected. Participants spoke about how patients could assess ‘aspects [of EPAs] around communication, relaying information, [and] patient comfort’ (P12, PM&R). During the creation of EPAs, they also noted how patient assessments could be collected and deliberated on whether patients could be members of the competence committee:

There's a Competency Committee to review the EPA … and there was lots of discussion about having a patient or general public representative on the Competency Committee. (P7, Gen. Surg.)

## 5 How Can Patients Contribute to Teaching and Assessing EPAs?

Participants explained that patients can (1) teach nontechnical aspects of EPAs through storytelling, (2) assess nontechnical skills and (3) contribute to the formative assessment of EPAs.

### 5.1 Teach Nontechnical Aspects of EPAs Through Storytelling

Participants believed that patients could contribute to teaching nontechnical skills encompassed within the EPAs through storytelling. They described patients' experiences of illness and healthcare as ‘beneficial’ (P5, OBGYN) and ‘valuable’ (P13, EMERG). One participant emphasized that learners would likely ‘walk away with something’ meaningful from such encounters (P6, OBGYN). Participants proposed that patients share their stories during academic half‐days and medical education conferences (P4, P5 and P6), as this would provide learners with opportunities to reflect on patient experiences and apply the lessons learned in clinical practice. Participants emphasized that patients' stories can leverage learners' empathy, and the reflective capacity required to master nontechnical skills:

If I could ask for one thing from my residents, it is that they gain a sense of empathy for what the patients go through. And, to me, that is the best piece of teaching or information that a resident could gain. So, I would not put any great expectations or pressure on a patient, other than to provide their personal experience of a case, or a treatment or an interaction. (P3, ANESTH)

### 5.2 Assess Nontechnical Skills

Participants emphasized that patients should assess EPAs focused on nontechnical skills. They explained that patients are the ‘most appropriate people to assess how an interaction went’ (P3, ANESTH) and how it made them feel. Patients have ‘really useful information … [that] faculty might not see’ (P19, Gen. Surg.). They gave specific examples of what patients could assess, including ‘how learners discussed care with patients’ (P3, ANESTH), ‘the timeliness of communication’ (P12, PM&R), and whether a learner was ‘too jargony’ (P16, EMERG) or ‘seemed aloof’ (P17, EMERG). They also believed patients should assess advocacy skills, such as how learners ‘worked on their behalf’ (P24, GIM). However, they noted that patient assessments of EPAs are not formally collected. To address this gap, participants suggested the development of ‘more patient‐oriented milestones that emphasize nontechnical skills’ (P6, OBGYN).

*Participants emphasized that patients should assess EPAs focused on nontechnical skills*.

### 5.3 Contribute to the Formative Assessment of EPAs

Participants believed that patients should contribute to the formative assessment of EPAs, explaining that patients should inform ‘the bigger picture of the assessment’ (P10, PM&R). Patient assessments offer ‘another data point’ (P24, GIM) that competence committees could use to contextualize a learner's performance. One participant noted, ‘If patients think that you're a terrible advocate or that you communicate badly, then we're going to have some stuff to talk about’ (P13, EMERG). They emphasized that patients offer insights into learners' performances of EPAs that should not be overlooked.

Yet, participants expressed concerns about the subjectivity of patient assessments, making them less suitable for summative assessments. They explained that ‘there's a lot of rigour that needs to go into creating a summative assessment’ (P1, Gen. Surg.), but patient assessments are ‘not black and white’ (P14, GIM). Participants suggested that if competence committees use patients' assessments for high‐stakes decisions on EPAs, they should be ‘amalgamated over a period of time’ (P25, ANESTH) and contextualized appropriately.

## 6 What Are the Perceived Barriers to Involving Patients in Teaching and Assessing EPAs?

The participants noted barriers related to (1) finding the ‘right’ patient, (2) a lack of time, (3) the risk of breaking patient anonymity, and (4) technology.

### 6.1 Finding the ‘Right’ Patient

Participants expressed that it is difficult to find the ‘right’ patient to teach or assess EPAs because patients must be in the right ‘state of mind’ (P6, OBGYN) or ‘headspace’ (P12, PM&R) to provide constructive information. For instance, ‘the sick patient [is] … just worried about getting better’ (P7, Gen. Surg.) and is ‘focused on being a patient’ (P23, EMERG), not on teaching and assessing EPAs. For example, participants explained that asking patients to provide assessments ‘when they are not at their best is fully inappropriate’ (P17, EMERG). Selecting patients to teach or assess EPAs must also be done ‘carefully’ (P9, Psychiatry) to avoid patients who ‘focus on extremes’ (P7, Gen. Surg.) or who use these opportunities to ‘complain’ (P3, ANESTH; P23, EMERG) or be ‘hurtful’ towards learners (P5, OBGYN and P8, EMERG). They suggested that patients be chosen to teach or assess EPAs who have ‘established rapport’ with the learner (P15, Rad. Onc.). Overall, participants acknowledged that the ‘right’ patient to teach or assess EPAs could provide constructive information, but they were uncertain how to identify these patients.

### 6.2 Lack of Time

Participants felt that there was insufficient time in the curriculum and clinical workflows to teach and assess EPAs. They explained that EPAs are ‘already packed’ (P4, OBGYN) with observations, and involving patients adds ‘another level of evaluation and work’ (P9, Psychiatry) for faculty physicians who are ‘busy’ (P8, EMERG), ‘tapped out’ (P20, EMERG), and have ‘minimal support for … this type of thing’ (P10, PM&R). They described patients teaching and assessing EPAs as potentially ‘disruptive to clinical workflow’ (P12, PM&R). As one participant observed, ‘[In] the current paradigm, where the emergency departments are all underwater, it's just not real‐life to ask patients for teaching and assessing EPAs’ (P17, EMERG).

To address these challenges, participants suggested that we need a ‘good way of building [patient involvement] as part of clinical interactions’ (P13, EMERG). Furthermore, ‘we should be looking at solutions for the overwhelming number of EPAs so that time is made for patient involvement’ (P19, Gen. Surg.).

### 6.3 Risk of Breaking Patient Anonymity

Participants said they do not know how to involve patients in teaching and assessing EPAs without breaking patient anonymity. They felt that patient anonymity is essential for ‘truthful’ (P5, OBGYN) and ‘honest’ (P16, EMERG) involvement. For instance, participants suggested that patient assessments be ‘de‐identified’ and ‘aggregated’ (P5, OBGYN) so that no individual patient could be identified. They also offered a ‘staff‐mediated’ approach (P3, ANESTH), where various members ask patients of the healthcare team to provide assessments of EPAs so that learners and/or medical educators are not privy to which patients were asked. Yet, participants struggled to identify an effective process to protect patient anonymity, especially regarding teaching, and they believed this was a significant barrier to involving patients.

### 6.4 Technological Barriers

Participants identified electronic EPA platforms (e‐platforms) as significant barriers to involving patients because e‐platforms ‘don't support patient information’ (P2, Psychiatry) and require faculty‐appointed or RCPSC login credentials, which limit accessibility for patients (P1, P3, P9, P14, P21 and P24). They explained that involving patients in teaching and assessing EPAs using current e‐platforms requires workarounds that are ‘extremely time‐consuming’ (P1, P3, P5 and P19). One participant described these workarounds as having to ‘come away from Elantra [the e‐platform] and somehow be connected back to [it]’ (P6, OBGYN). Despite acknowledging that e‐platforms ‘need tweaking’ (P19, Gen. Surg.), participants expressed uncertainty about effectively re‐designing e‐platforms to support patient involvement.

## 7 Discussion

This study explored faculty members' perspectives on involving patients in teaching and assessing EPAs. While participants acknowledged the value of involving patients, they alluded to many challenges and a lack of clear guidance on involving them within the current CBD context. They also described how the existing EPAs within the CBD context still characterize patients as ‘subjects for teaching’, which reflects a traditional and paternalistic perspective [19]. While some participants viewed patients as potential educators, particularly of nontechnical EPA components, a broader cultural shift is necessary.

*While some participants viewed patients as potential educators, particularly of nontechnical EPA components, a broader cultural shift is necessary*.

The findings indicate the need for guidelines to support such patient involvement [13, 14]. These guidelines could address, for example, the participants' suggestions for creating context‐specific patient engagement criteria tailored to each specialty and patient population. They could also address the participants' concerns about when and how to involve patients to ensure they are emotionally or physically prepared to participate [15]. Ultimately, these guidelines could help normalize patient involvement and encourage early and sustained engagement [20].

*The findings indicate the need for guidelines to support patient involvement*.

Moreover, participants noted that EPAs are saturated with observational requirements and that clinical environments may not easily accommodate additional patient roles. They described how the pressure for learner‐centered CBD [5, 8] and an efficient healthcare system [21, 22] leaves minimal opportunities for patient involvement in teaching and assessing EPAs. Given this reality, future research should review which EPAs and their associated milestones are suitable for patient input [14]. This patient involvement may help distribute responsibilities more equitably between faculty members and patients and relieve some of the pressure the participants described. Developing dedicated patient dashboards could also address some participants' noted barriers [23]. These dashboards would make it easier for patients to become involved in teaching and assessing EPAs.

However, it is important to note the study limitations. First, the findings reflect the opinions of faculty members involved with EPAs. Future research should explore patients' perspectives on this topic. Such research could examine patients' comfort levels, roles and expectations in teaching and assessing EPAs. It could also gather patients' perspectives on how the barriers identified in the present study should be addressed. Second, only eight RCPSC specialties were included in the study. Exploring patient involvement in other specialties may reveal new challenges and solutions to involving patients in teaching and assessing EPAs. Finally, this study did not critically examine the EPAs themselves. Future research should explore the structure and assumptions of the EPAs and determine where revisions are needed to facilitate patient involvement.

- Next steps to support patient involvement in teaching and assessing EPAs within CBD
- Develop clear guidelines, tailored to specific specialties and patient populations, for involving patients in teaching and assessing EPAs.
- Ensure guidelines address when and how to involve patients, including considerations for their emotional and physical readiness.
- Promote a cultural shift in medical education that recognizes and supports patients as active contributors and potential educators within CBD.
- Normalize and integrate patient engagement early and consistently throughout CBD.
- Identify which EPAs and milestones are most appropriate for meaningful and feasible patient input.
- Address practical and system‐level barriers, such as time constraints and clinical efficiency demands, to make space for patients in teaching and assessing EPAs.
- Develop and implement patient‐facing technological tools to support their participation in EPA‐related activities.
- Conduct research to understand patients' perspectives, including their comfort levels, expectations, and perceived roles in teaching and assessing EPAs.
- Critically review the structure and assumptions of EPAs to assess whether revisions are needed to facilitate patient involvement.

## 8 Conclusion

This study explored faculty members' perspectives regarding patient involvement in teaching and assessing EPAs. The findings highlight the need for clear guidance on improving patient involvement in these fundamental CBD processes. Addressing such involvement and lack thereof is not merely an academic exercise but a pivotal step towards elevating the quality, authenticity, and patient‐centeredness of medical education within CBD.

## Author Contributions

**Holly Adam:** conceptualization, writing – original draft, writing – review and editing, formal analysis. **Kaylee Eady:** writing – review and editing, conceptualization, supervision. **Katherine A. Moreau:** conceptualization, writing – original draft, writing – review and editing, formal analysis, supervision, conceptualization, writing – review and editing, writing – original draft.

## Ethics Statement

This study received REB approval from the University of Ottawa (REB # S‐02‐22‐7832) on 20 April 2022.

## Conflicts of Interest

The authors declare no conflicts of interest.

## Data Availability

The data that support the findings of this study are available on request from the corresponding author. The data are not publicly available due to privacy or ethical restrictions.
